# Comparison of PRISMA Data with Model Simulations, Hyperion Reflectance and Field Spectrometer Measurements on ‘Piano delle Concazze’ (Mt. Etna, Italy)

**DOI:** 10.3390/s20247224

**Published:** 2020-12-17

**Authors:** Vito Romaniello, Malvina Silvestri, Maria Fabrizia Buongiorno, Massimo Musacchio

**Affiliations:** Istituto Nazionale di Geofisica e Vulcanologia (INGV), 00143 Roma, Italy; malvina.silvestri@ingv.it (M.S.); fabrizia.buongiorno@ingv.it (M.F.B.); massimo.musacchio@ingv.it (M.M.)

**Keywords:** PRISMA mission, hyperspectral data, Mt. Etna

## Abstract

In this work, we compare first acquisitions from the ASI-PRISMA (Agenzia Spaziale Italiana-PRecursore IperSpettrale della Missione Applicativa) space mission with model simulations, past data acquired by the Hyperion sensor and field spectrometer measurements. The test site is ‘Piano delle Concazze’ (Mt. Etna, Italy), suitable for calibration purposes due to its homogeneity characteristics. The area measures at about 0.2 km^2^ and is composed of very homogeneous trachybasalt rich in plagioclase and olivine. Three PRISMA acquisitions, achieved on 31 July and 8 and 17 August 2019, are analyzed. Firstly, spectral profiles of PRISMA top of atmosphere (TOA) radiance are compared with MODerate resolution atmospheric TRANsmission (MODTRAN) simulations. The Pearson correlation coefficient is equal to 0.998 and 0.994 for VNIR (Visible and Near InfraRed) and SWIR (Short-Wave InfraRed) spectral ranges, respectively. PRISMA radiance overestimates values simulated by MODTRAN for all considered days, showing a mean bias of +5.22 and of +0.91 Wm^−2^sr^−1^µm^−1^ for VNIR and SWIR, respectively. The relative mean difference between reflectance values estimated by PRISMA and Hyperion, on the test area, is around +19%, despite the great difference in time acquisition (up to 19 years); PRISMA slightly overestimates Hyperion reflectance with an absolute mean difference of about +0.0083, within the variability of Hyperion acquisitions of ±0.0250 (corresponding to ±2 standard deviation). Finally, FieldSpec measurements also confirm the great quality of PRISMA reflectance estimations. The absolute mean difference results are around +0.0089 (corresponding to a relative error of about +21%). In the study, we investigate only the lower values of reflectance characterizing the test site. A more complete evaluation of PRISMA performances needs to consider other test sites with different optical characteristics.

## 1. Introduction

Spectral imaging is a data-intensive method that samples image data on the electromagnetic spectrum. The information retrieved through spectral imagery is crucial for a wide range of earth sciences applications, such as for geology [1], agriculture [2] and water management aspects [3,4], inland and coastal water monitoring [5], gas emission retrieval [6] and fire detection [7]. When the reflected energy is sensed within multiple wavelength bands (<20), the imaging process is referred to as multispectral (e.g., the Landsat mission). Retrieving data at higher bands with a narrower wavelength (>20) refers to hyperspectral imaging (e.g., the Airborne Visible/InfraRed Imaging Spectrometer (AVIRIS) [8]).

Hyperspectral images allow to distinguish different materials on the Earth’s surface, recognizing its spectral signatures; thus, they are a powerful geology tool, allowing to detect several minerals with significant implications for mining activities [9] and volcanic mapping [10]. Hyperspectral sensors typically collect 200 or more contiguous bands [11], enabling the reconstruction of vegetation signatures [12]. Agriculture studies can benefit significantly from hyperspectral imaging analysis for monitoring crop health status [13].

Hyperspectral remote sensing techniques for coastal water analysis have been developed for more than three decades [11,14]. For this type of application, the availability of a continuous spectrum makes algorithms more effective in a wide variety of waters with varying water column depths and leads to a better retrieval of a large number of properties [15]. Moreover, hyperspectral remote sensing allows the identification and quantification of gas emissions in the atmosphere and pollutants [16]. Images acquired during an airborne campaign by AVIRIS instrument over the Puʻu ʻŌʻō vent (situated at the Kilauea East Rift zone, Hawaii) allowed for producing carbon dioxide maps of plumes emitted from the crater [17]. Moreover, the shortwave infrared region of the e.m. spectrum (in the range 1.4–2.5 µm), generally employed in hyperspectral sensors, can include significant emitted radiance from fires [18] and can be used for detecting flaming combustion [19].

The Italian Space Agency launched a hyperspectral imaging platform, PRecursore IperSpettrale della Missione Applicativa (PRISMA), in 2019 [20]. While some studies have tried to evaluate PRISMA data for specific applications (e.g., [15] for waterbodies; [21] for topsoil properties), an evaluation of PRISMA radiometric performance and its level of accuracy in retrieving surface reflectance has not been yet established. Evaluation of satellite acquisitions requires proximal spectral measurements (e.g., using field hyperspectral radiometers) [22,23,24].

In this paper, we tested the accuracy of PRISMA acquisitions in the range 0.4–2.5 µm with reference to past measurements from the Hyperion mission [25] (launched on 21 November 2000). The considered test site is a flat area on the Mt. Etna volcano called ‘Piano delle Concazze’ (PdC), for which the availability of field spectrometer measurements, collected during past surveys, allowed for performing the assessments with proximal measurements.

## 2. Test Site Description

In the convergence margin between the African and European Plates, Mt. Etna is situated on the eastern coast of Sicily, Italy [26]. The Etna area is characterized by a complex geodynamic framework ranging from a regional N–S compressive regime under the volcanic pile [27], due to the plate collision, co-existing with a WNW–ESE extensional regime, associated with the dynamics of the Malta Escarpment [28].

**Figure 1:**
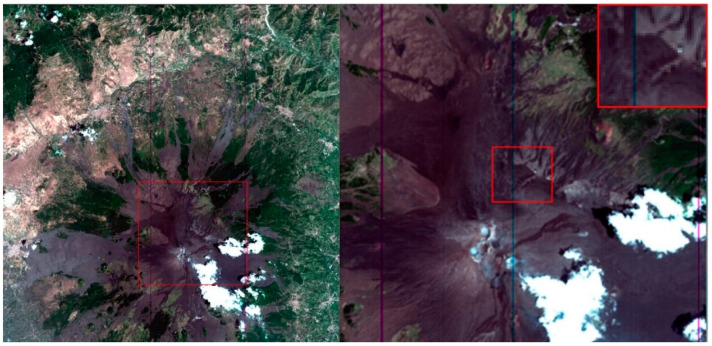
RGB (Red Green Blue) image of Mt. Etna and zoom on Piano delle Concazze (PdC) by PRecursore IperSpettrale della Missione Applicativa (PRISMA) acquisition on 31 July 2019.

**Figure 2:**
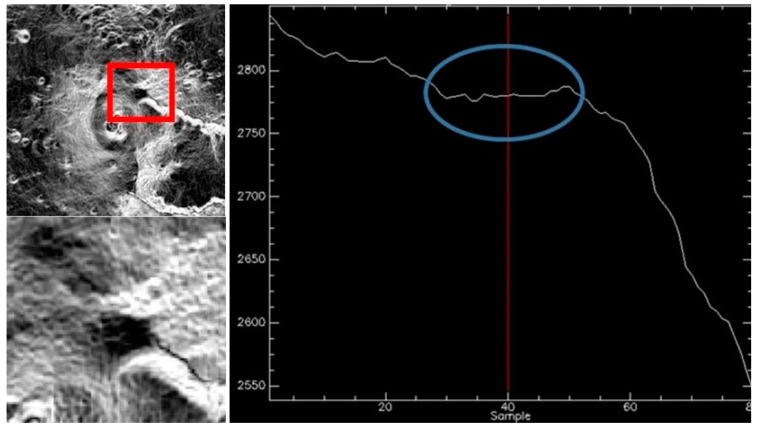
Slope of the PdC area: horizontal profile (**left**); vertical profile in meters a.s.l. (**right**).

The volcano conduit is an open system, constantly filled with magma and showing continuous activity. In recent years, after the effusive flank eruption of May 2008 to July 2009, there was an extraordinary period of explosive activity at Etna volcano, with 45 lava fountains emitted from the New South East crater (NSEC) during 2011–2015 [29]. Sequences of lava fountains took place in December 2015 and May 2016 from the main central crater Voragine (VOR) [30,31] and a huge eruption occurred on December 24, 2018 [32,33]. These volcanic activities have not affected the test site considered in the present work. The PdC site is located on the north-east side of the Mt. Etna volcano: latitude = 37.766 deg, longitude = 15.013 deg (see red box in the Figure 1). PdC is a large area dominated by the North East crater and bound by the rim of the wide depression of the Valle del Bove; it is composed of very homogeneous trachybasalt rich in plagioclase and olivine and characterized by a flat geometry with an altitude between 2775 and 2790 m (a.s.l.) (see Figure 2). The PdC area is about 0.2 km^2^ and is easily identifiable by satellite data with a spatial resolution of 30 m or finer. The PdC area was selected for the above reasons and for the availability of past field campaign measurements in situ.

## 3. Satellite Dataset, Field Measurements and Simulated Data

The PRISMA dataset considered in this work includes 3 images on the test area acquired from July to August 2019 (see also Table 1); these satellite acquisitions are clean of clouds and snow on the surface. The MODerate resolution atmospheric TRANsmission (MODTRAN) [34] simulations were performed by using version 4.0 of the code and considering atmospheric profiles (temperature, pressure and humidity) near-synchronous with PRISMA acquisitions; model runs employed, as an input parameter, the reflectance spectrum measured at the test site. Hyperion images acquired in the period 2001–2009 (19 images overall) are considered in the comparison; reflectance values obtained by Hyperion acquisitions are compared with PRISMA reflectance estimations. Moreover, the spectrum measured by the spectrometer FieldSpec ASD in the range of 0.4–2.5 µm, at PdC during the July 2003 campaign, is considered in the comparison analyses.

### 3.1. PRISMA Data Description

**Figure 3:**
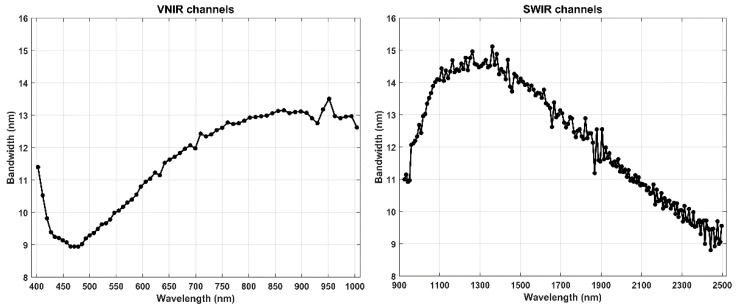
Channels and bandwidth of the PRISMA sensor: VNIR on left and SWIR on right.

The PRISMA satellite, launched on 22 March 2019, holds a hyperspectral and panchromatic payload which is able to acquire images with a worldwide coverage [35]. The hyperspectral camera works in the spectral range of 0.4–2.5 µm, with 66 and 173 channels in the VNIR (Visible and Near InfraRed) and SWIR (Short-Wave InfraRed) regions, respectively. The average spectral resolution is less than 10 nm on the entire range with an accuracy of ±0.1 nm. VNIR and SWIR channels are overlapped between 930 and 1000 nm and the FWHM (Full Width at Half Maximum) is in the range of 9–15 nm (see Figure 3).

The ground sampling distance of PRISMA images is about 5 and 30 m for panchromatic and hyperspectral camera, respectively. In the present study, we employed only hyperspectral data and, in particular, the Level-1 (top of atmosphere (TOA) radiance) and the Level-2D (reflectance at ground) delivered by the Italian Space Agency by through the PRISMA portal [36].

### 3.2. MODTRAN Parameters for Simulations

In order to simulate the radiance emitted from PdC, as well as seen from PRISMA, the MODTRAN^TM^ code [37,38] was used for the spectral region in the range of 0.4–2.5 µm. This code offers the possibility to calculate the atmospheric contribution to the radiance emitted by modeling atmospheric inputs, surface reflectance parameters and path geometry. MODTRAN has the capability to combine the main effects in the atmosphere as the absorption/emission, scattering, surface reflection and emission.

Table 2 reports the main input parameters for MODTRAN simulations. The atmospheric vertical profiles of temperature, pressure and humidity, collected by radiosonde up to 25 km, were downloaded from the free available repository of the University of Wyoming [39]. The MODTRAN output considered in the following analyses is the top of atmosphere (TOA) radiance.

### 3.3. Hyperion Data Description

The Hyperion instrument was launched on board the Earth Observing-1 (EO-1) satellite on 21 November 2000, which was decommissioned on March 2017. Hyperion collected 220 spectral channels, ranging from 0.357 to 2.576 µm, with a 10-nm bandwidth; the spatial resolution is of 30 m for all bands. In the present work, the Hyperion dataset includes 19 acquisitions, achieved from 2001 to 2009, at the PdC test site. The Hyperion reflectance was obtained by means of the CIRILLO method applied to TOA radiance images [40].

### 3.4. FieldSpec Data Description

**Figure 4:**
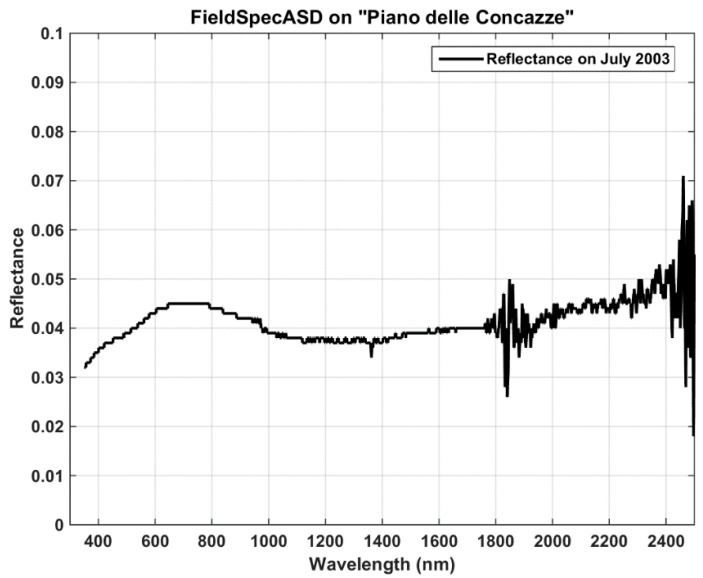
FieldSpec spectrum collected at ‘PdC’ during the July 2003 campaign.

The reflectance spectrum, in the range of 0.4–2.5 µm, was acquired with a field spectroradiometer (Fieldspec ASD) during a measurement campaign at the PdC site in July 2003. This instrument has a spectral resolution of 3 nm at 700 nm and 10 nm at 1400–2100 nm and a sampling step of 1.4 nm at 350–1050 nm and 2 nm at 1000–2500 nm. During the field campaign of July 2003, measurements of surface reflectance were collected between 10:00 and 12:00 (local time), synchronous with Hyperion acquisition [23]. In Figure 4, the reflectance measured at ‘PdC’, characterized by very low values in the range 0.03–0.05, shows the presence of fine tephra and ash deposits.

## 4. Results of Comparisons

In this chapter, results of comparisons are reported. Firstly, TOA radiances are obtained by MODTRAN simulation runs; secondly, the reflectance spectra estimated by Hyperion and measured by the FieldSpec spectrometer are considered. Specifically, analyses of radiances are considered separately for VNIR and SWIR spectral ranges, while comparisons of reflectance values are performed on the entire spectra in the range 0.4–2.5 µm.

### 4.1. PRISMA vs. MODTRAN Simulations: TOA Radiance Comparison

**Figure 5:**
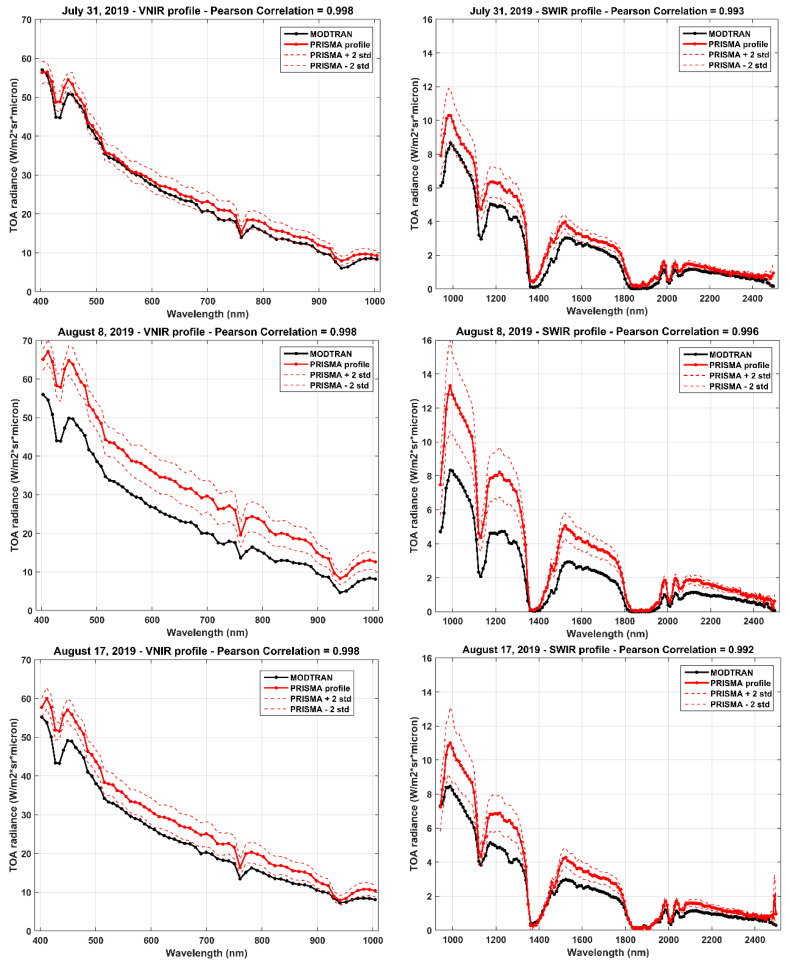
Spectral profiles of PRISMA (red line) and MODTRAN simulations (black line) for VNIR (on **left**) and SWIR (on **right**); acquisitions and simulations on 31 July, 8 and 17 August 2019.

Spectra profiles of PRISMA and model simulations are reported for VNIR and SWIR spectral ranges and for the three considered days (see Figure 5).

**Figure 6:**
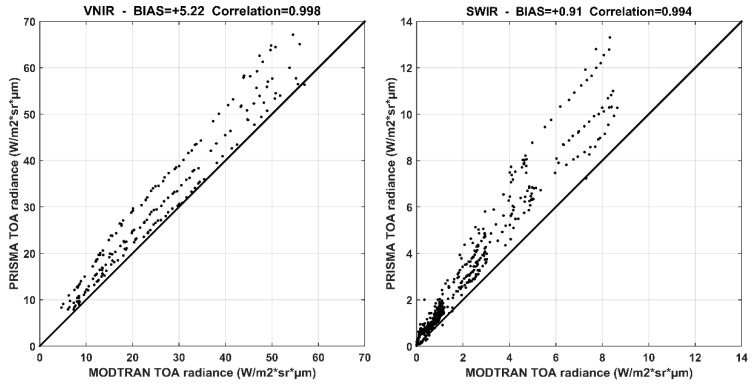
Scatter plots of radiance values for VNIR (on **left**) and SWIR (on **right**) considering all three days’ data.

The Pearson correlation coefficient is equal to 0.998 and 0.994 for the VNIR and SWIR spectral ranges, respectively. Moreover, PRISMA TOA radiance overestimates model values for all three days of measurements (see scatter plots of comparisons in Figure 6).

Regarding the VNIR spectral range, the overestimation is about 2.61, 8.80 and 4.25 Wm^−2^sr^1^µm^−1^, for the three considered days, respectively (see Table 3); only for the July 31 measurement, the simulated profile is included in the ±2 standard deviation range. Furthermore, for the SWIR spectral range, the comparison results show very similar behaviors: mean PRISMA values overestimate MODTRAN simulations by 0.64, 1.37 and 0.72 Wm^−2^sr^−1^µm^−1^ (see Table 3). Differences in the comparison may be due to sensor calibration errors and uncertainties related to atmospheric conditions [41,42].

### 4.2. PRISMA vs. Hyperion: Reflectance Comparison

**Figure 7:**
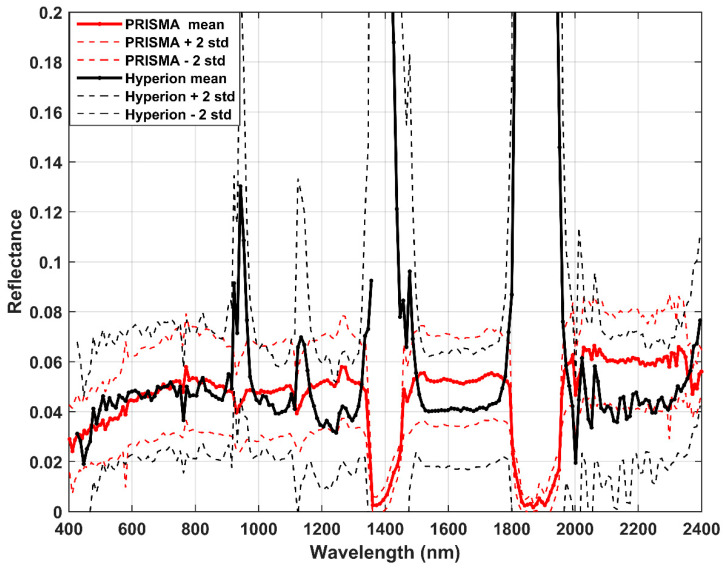
Profiles of PRISMA (red) and Hyperion (black).

Reflectance values estimated by PRISMA for the three dates are compared with past measurements by Hyperion in the period 2001–2009. Specifically, the PRISMA mean values are obtained from the average of the three acquisitions, and the standard deviation is also calculated and reported. The comparison results show an overestimation of PRISMA values which are included in the interval of ±2 standard deviation, representing the variability of Hyperion values. Figure 7 shows four picks at 941, 1137, 1415 and 1875 nm due to the water absorptions not completely compensated in Hyperion images.

With the aim to compare results, five spectral sectors are considered excluding the water absorption regions (see Table 4).

PRISMA reflectance overestimates Hyperion in all sectors except the first one; the overall main difference is about 0.0083 and is contained in the variability of Hyperion values, corresponding to ±0.0250 (equivalent to ±2 standard deviation). The relative overestimation results at about 19%, but this parameter has little statistical significance because we are probing only very low values of reflectance.

The timing of satellite measurements is very different and the ideal comparison would be for synchronous measurements. Unfortunately, this was not possible because the EO-1 Hyperion mission was decommissioned in March 2017 and the PRISMA mission represents the natural successor of Hyperion. The comparison of reflectance is, however, valid (within a certain error) for two main reasons: first, the surface has never been involved in new volcanic eruptions, although small changes in the surface roughness probably occurred; second, the contribution of atmosphere was removed from satellite images, at the same time of acquisitions, to obtain reflectance products. Therefore, the comparison is not affected by atmospheric conditions or great surface changes.

### 4.3. PRISMA vs. FieldSpec: Reflectance Comparison

**Figure 8:**
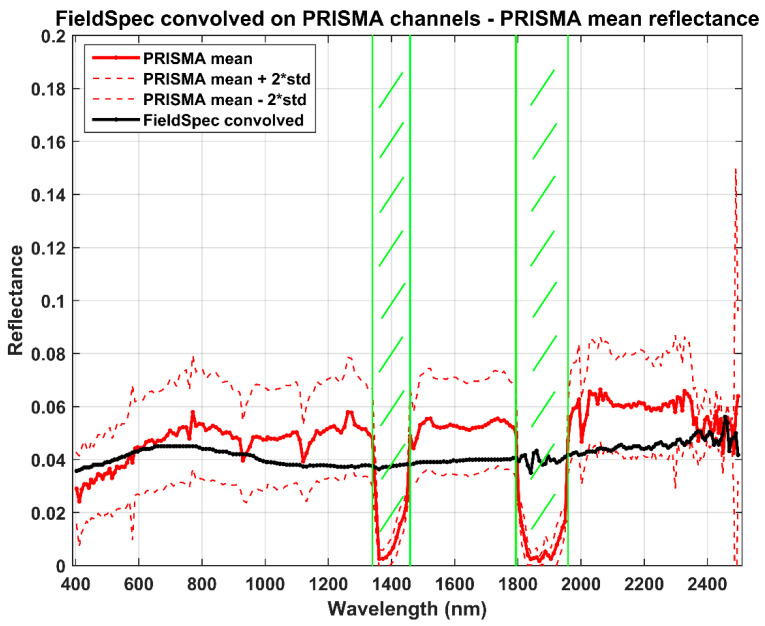
Reflectance profiles of PRISMA (red) and FieldSpec (black).

**Figure 9:**
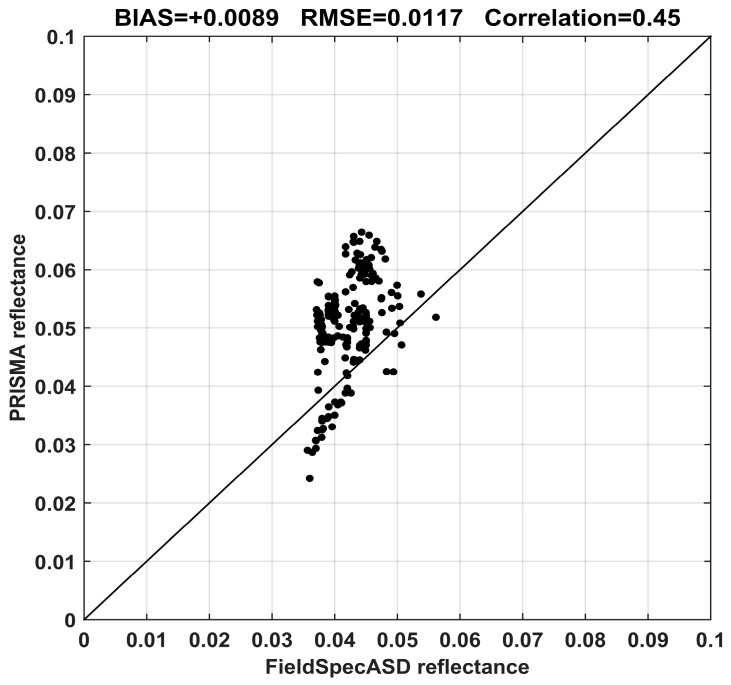
Scatter plot of PRISMA and FieldSpec reflectance values.

Lastly, the PRISMA reflectance values are compared with the measurement by the FieldSpec ASD in July 2003 [43]. The ground measurement, at very high spectral resolution (in the order of a few meters), is convolved on Gaussian response functions centered at PRISMA channels. Regions around 1415 and 1875 nm are excluded from the comparison, being strongly affected by water absorption. Results of the comparison are shown in Figure 8 and Figure 9.

The mean reflectance value estimated by PRISMA is about 0.0511 with respect to the FieldSpec mean value of 0.0422. Despite the big difference in time acquisition between the two measurements (2003 and 2020 for ground and space measurements, respectively), the absolute mean error is +0.0089 (corresponding to a relative error of about +21%); this also confirms the great homogeneity of the PdC test site in terms of time and space. The correlation between the two sets of measurements is around 0.45, showing a moderate agreement; this depends on atmospheric gas absorptions not removed in satellite acquisitions.

## 5. Conclusions

First, acquisitions from the PRISMA space mission were compared with MODTRAN model simulations, past data acquired by the Hyperion sensor in the period 2001–2009 and field spectrometer measurements. The considered test site is ‘Piano delle Concazze’ (Mt. Etna, Italy), which is a flat and homogeneous area of about 0.2 km^2^. The site is located at 2780 m of altitude in a position not covered by the degassing plume most of the time, thus making it suitable for calibration purposes; moreover, past Hyperion acquisitions demonstrated a high spectral homogeneity.

Spectra profiles of PRISMA TOA radiance were compared with MODTRAN simulations for the three days considered in this work. The correlation is very high for both VNIR and SWIR spectral ranges, resulting greater than 0.998 and 0.994, respectively. PRISMA radiance overestimated model values for the three days considered, showing a mean bias of +5.22 Wm^−2^sr^−1^µm^−1^ (about +20%) and of +0.91 Wm^−2^sr^−1^µm^−1^ (about +43%) for the two different spectral ranges. The biggest difference with modeled radiance values out of the PRISMA estimations’ variability (out of ±2 SD) was for the August 8 acquisition; this case needs further investigation.

The agreement between reflectance values estimated by PRISMA and Hyperion on the PdC area was contained in a relative mean difference of about 19%, despite the great difference in time acquisition (up to 19 years). PRISMA slightly overestimates Hyperion reflectance, with an overall mean difference of about +0.0083, within the variability of Hyperion acquisitions of ±0.0250 (corresponding to ±2 SD). The timing of satellite measurements is very different, but the comparison of reflectance is, however, valid, because the atmospheric contribution to imagery was removed and small changes in surface roughness occurred.

Finally, the FieldSpec measurements also confirmed the great quality of PRISMA reflectance estimations. The relative main difference was about +0.0089 (corresponding to a relative error of about +21%).

The evaluation of PRISMA performance, in terms of reflectance estimation, is not complete in the present work because we considered only low levels of reflectance values. A more complete evaluation needs to consider other test sites, with different optical characteristics, and field campaigns synchronous with PRISMA satellite passages. The Italian National Institute of Geophysics and Volcanology (INGV) is also planning a new field campaign at PdC to measure ground reflectance and better evaluate aerosol characteristics and vertical atmospheric profiles.

## Figures and Tables

**Table 1:** Dataset used for comparison purposes.

	PRISMA	MODTRAN (Simulations)	Hyperion	FieldSpec ASD
Radiance (TOA)	31 July 2019 8 August 2019 17 August 2019	Point simulations synchronous with PRISMA	n/a	n/a
Reflectance	31 July 2019 8 August 2019 17 August 2019	n/a	Acquisitions from 2001 to 2009	Field campaign in July 2003

**Table 2:** Main input parameters for MODerate resolution atmospheric TRANsmission (MODTRAN) model runs.

MODTRAN Parameter	Value
Surface albedo	Reflectance at ground acquired with the FieldSpec ASD spectrometer at PdC
Surface temperature	283 K
Geographical-seasonal model atmosphere	Radiosonde profiles acquired from Trapani (Sicily) station on 31 July, 8 and 17 August 2019; Mid-latitude summer (above 25 km)
Altitude of surface (a.s.l.)	2700 m (PdC mean altitude)
Initial zenith angle as measured from PdC	178 deg
Spectral range	0.4–2.5 µm (4000–25,000 cm^−1^)
Sun zenith angle	24°

**Table 3:** Mean TOA radiance values from PRISMA and model simulations.

Date	Model Simulations VNIR	PRISMA VNIR	Model Simulations SWIR	PRISMA VNIR
31 July 2019	25.53	27.14	2.11	2.75
8 August 2019	24.84	33.64	1.94	3.31
17 August 2019	24.83	29.08	2.19	2.91

**Table 4:** Mean reflectance values from Hyperion and PRISMA in the considered spectral sectors; the absolute differences are also reported.

Spectral Range (nm)	Hyperion	PRISMA	Absolute Difference PRISMA-Hyperion
Sector 1: 427.1–908.9	0.0448	0.0438	−0.0010
Sector 2: 972.9–1109.8	0.0438	0.0484	0.0046
Sector 3: 1163.5–1328.1	0.0387	0.0522	0.0135
Sector 4: 1501.8–1774.9	0.0427	0.0531	0.0104
Sector 5: 1975.8–2364.4	0.0445	0.0606	0.0161

## References

[1] Lorenz S., Zimmermann R., Gloaguen R. (2017). The Need for Accurate Geometric and Radiometric Corrections of Drone-Borne Hyperspectral Data for Mineral Exploration: MEPHySTo—A Toolbox for Pre-Processing Drone-Borne Hyperspectral Data. Remote. Sens..

[2] Niyogi D., Jamshidi S., Smith D., Kellner O. (2020). Evapotranspiration Climatology of Indiana, USA Using In-Situ and Remotely Sensed Products. J. Appl. Meteorol. Clim..

[3] Jamshidi S., Zand-Parsa S., Jahromi M.N., Niyogi D. (2019). Application of A Simple Landsat-MODIS Fusion Model to Estimate Evapotranspiration over A Heterogeneous Sparse Vegetation Region. Remote. Sens..

[4] Jamshidi S., Zand-Parsa S., Pakparvar M., Niyogi D. (2019). Evaluation of Evapotranspiration over a Semiarid Region Using Multiresolution Data Sources. J. Hydrometeorol..

[5] Chander S., Gujrati A., Krishna A.V., Sahay A., Singh R. (2020). Remote sensing of inland water quality: A hyperspectral perspective. Hyperspectral Remote Sensing.

[6] Hulley G.C., Duren R.M., Hopkins F.M., Hook S.J., Vance N., Guillevic P., Johnson W.R., Eng B.T., Mihaly J.M., Jovanovic V.M. (2016). High spatial resolution imaging of methane and other trace gases with the airborne Hyperspectral Thermal Emission Spectrometer (HyTES). Atmos. Meas. Tech..

[7] Bagheri N., Mohamadi-Monavar H., Azizi A., Ghasemi A. (2017). Detection of Fire Blight disease in pear trees by hyperspectral data. Eur. J. Remote Sens..

[8] National Aeronautics and Space Administration (NASA) (2011). AVIRIS Home Page. http://aviris.jpl.nasa.gov/.

[9] Ramakrishnan D., Bharti R. (2015). Hyperspectral remote sensing and geological applications. Curr. Sci..

[10] Li L., Solana C., Canters F., Chan J.C.-W., Kervyn M. (2015). Impact of Environmental Factors on the Spectral Characteristics of Lava Surfaces: Field Spectrometry of Basaltic Lava Flows on Tenerife, Canary Islands, Spain. Remote. Sens..

[11] Govender M., Chetty K., Bulcock H.H. (2009). A review of hyperspectral remote sensing and its application in vegetation and water resource studies. Water SA.

[12] Hernández-Clemente R., Hornero A., Mottus M., Penuelas J., González-Dugo V., Jiménez J.C., Suárez L., Alonso L., Zarco-Tejada P.J. (2019). Early Diagnosis of Vegetation Health From High-Resolution Hyperspectral and Thermal Imagery: Lessons Learned From Empirical Relationships and Radiative Transfer Modelling. Curr. For. Rep..

[13] Jamshidi S., Zand-parsa, Niyogi D. (2020). Assessing Crop Water Stress Index for Citrus Using In-Situ Measurements, Landsat, and Sentinel-2 Data. Int. J. Remote Sens..

[14] Flores-Anderson A.I., Griffin R., Dix M., Romero-Oliva C.S., Ochaeta G., Skinner-Alvarado J., Moran M.V.R., Hernandez B., Cherrington E., Page B. (2020). Hyperspectral Satellite Remote Sensing of Water Quality in Lake Atitlán, Guatemala. Front. Environ. Sci..

[15] Giardino C., Bresciani M., Braga F., Fabbretto A., Ghirardi N., Pepe M., Gianinetto M., Colombo R., Cogliati S., Ghebrehiwot S. (2020). First Evaluation of PRISMA Level 1 Data for Water Applications. Sensors.

[16] Sabbah S., Rusch P., Gerhard J.-H., Harig R. (2010). An infrared hyperspectral sensor for remote sensing of gases in the atmosphere. Remote Sensing.

[17] Spinetti C., Carrère V., Buongiorno M.F., Sutton A.J., Elias T. (2008). Carbon dioxide of PuuOo volcanic plume at Kilauea retrieved by AVIRIS hyperspectral data. Remote. Sens. Environ..

[18] Veraverbeke S., Dennison P., Gitas I.Z., Hulley G.C., Kalashnikova O.V., Katagis T., Kuai L., Meng R., Roberts D.A., Stavros E.N. (2018). Hyperspectral remote sensing of fire: State-of-the-art and future perspectives. Remote. Sens. Environ..

[19] Dennison P., Roberts D.A. (2009). Daytime fire detection using airborne hyperspectral data. Remote. Sens. Environ..

[20] Loizzo R., Guarini R., Longo F., Scopa T., Formaro R., Facchinetti C., Varacalli G. (2018). Prisma: The Italian Hyperspectral Mission. Proceedings of the IGARSS 2018–2018 IEEE International Geoscience and Remote Sensing Symposium.

[21] Casa R., Pignatti S., Pascucci S., Ionca V., Mzid N., Veretelnikova I. (2020). Assessment of PRISMA imaging spectrometer data for the estimation of topsoil properties of agronomic interest at the field scale. EGU General Assembly Conference Abstracts.

[22] Colini L., Spinetti C., Doumaz F., Amici S., Ananasso C., Buongiorno M.F., Cafaro P., Caltabiano T., Curci G., D’Andrea S. 2012 hyperspectral airborne campaign on Etna: Multi data acquisition for ASI-PRISMA project. Proceedings of the 2013 IEEE International Geoscience and Remote Sensing Symposium–IGARSS.

[23] Buongiorno M.F., Amici S., Colini L., Di Stefano G., Doumaz F., Lombardo V., Caltabiano T. (2008). Etna 2003 Field Campaign: Calibration and Validation of spaceborne and airborne Instruments for volcanic Applications. Quad. Geofis..

[24] Teillet P., Fedosejevs G., Gauthier R., O’Neill N., Thome K., Biggar S., Ripley H., Meygret A. (2001). A generalized approach to the vicarious calibration of multiple Earth observation sensors using hyperspectral data. Remote. Sens. Environ..

[25] USGS Website for the the Earth Observing-1 Mission and Hyperion Sensor Description. https://www.usgs.gov/centers/eros/science/usgs-eros-archive-earth-observing-one-eo-1-hyperion?qt-science_center_objects=0#qt-science_center_objects.

[26] Gvirtzman Z., Nur A. (1999). The formation of Mount Etna as the consequence of slab rollback. Nat. Cell Biol..

[27] Cocina O., Neri G., Privitera E., Spampinato S. (1997). Stress tensor computations in the Mount Etna area (Southern Italy) and tectonic implications. J. Geodyn..

[28] Hirn A., Nicolich R., Gallart J., Laigle M., Cernobori L. (1997). ETNASEIS Scientific Group Roots of Etna volcano in faults of great earthquakes. Earth Planet. Sci. Lett..

[29] Corradini S., Guerrieri L., Lombardo V., Merucci L., Musacchio M., Prestifilippo M., Scollo S., Silvestri M., Spata G., Stelitano D. (2018). Proximal Monitoring of the 2011–2015 Etna Lava Fountains Using MSG-SEVIRI Data. Geosciences.

[30] Aloisi M., Jin S., Pulvirenti F., Scaltrito A. (2017). The December 2015 Mount Etna eruption: An analysis of inflation/deflation phases and faulting processes. J. Geodyn..

[31] Bonaccorso A., Calvari S. (2017). A new approach to investigate an eruptive paroxysmal sequence using camera and strainmeter networks: Lessons from the 3–5 December 2015 activity at Etna volcano. Earth Planet. Sci. Lett..

[32] Calvari S., Bilotta G., Bonaccorso A., Caltabiano T., Cappello A., Corradino C., Del Negro C., Ganci G., Neri M., Pecora E. (2020). The VEI 2 Christmas 2018 Etna Eruption: A Small But Intense Eruptive Event or the Starting Phase of a Larger One?. Remote Sens..

[33] Corradini S., Guerrieri L., Stelitano D., Salerno G., Scollo S., Merucci L., Prestifilippo M., Musacchio M., Silvestri M., Lombardo V. (2020). Near Real-Time Monitoring of the Christmas 2018 Etna Eruption Using SEVIRI and Products Validation. Remote Sens..

[34] Website for the MODTRAN Model Description. http://modtran.spectral.com/modtran_index.

[35] Pignatti S., Palombo A., Pascucci S., Romano F., Santini F., Simoniello T., Umberto A., Vincenzo C., Acito N., Diani M. The PRISMA hyperspectral mission: Science activities and opportunities for agriculture and land monitoring. Proceedings of the 2013 IEEE International Geoscience and Remote Sensing Symposium-IGARSS.

[36] PRISMA Website for Data Download. https://prisma.asi.it.

[37] Berk A., Anderson G., Acharya P., Bernstein L., Muratov L., Lee J., Fox M., Adler-Golden S., Chetwynd J., Hoke M. (2006). MODTRANTM 5: 2006 update. Proc. SPIE Int. Soc. Opt Eng..

[38] Berk A., Conforti P., Kennett R., Perkins T., Hawes F., Van Den Bosch J. MODTRAN6: A major upgrade of the MODTRAN radiative transfer code. Proceedings of the 2014 6th Workshop on Hyperspectral Image and Signal Processing: Evolution in Remote Sensing (WHISPERS).

[39] University of Wyoming Website for the Atmospheric Profiles Download. http://weather.uwyo.edu/upperair/sounding.html.

[40] Musacchio M., Amici S., Teggi S., Pompilio L., Sgavetti M., Buongiorno M.F. (2007). Una nuova procedura per le Correzioni Atmosferiche: Applicazione sulla Solfatara di Pozzuoli. Riv. Ital. Telerilevamento.

[41] Yao B., Liu C., Teng S., Bi L., Zhang Z., Zhang P., Sohn B.-J. (2020). An accurate and efficient radiative transfer model for simulating all-sky images from Fengyun satellite radiometers. Sci. China Earth Sci..

[42] Ham S.-H., Sohn B., Yang P., Baum B.A. (2009). Assessment of the Quality of MODIS Cloud Products from Radiance Simulations. J. Appl. Meteorol. Clim..

[43] Spinetti C., Mazzarini F., Casacchia R., Colini L., Neri M., Behncke B., Salvatori R., Buongiorno M.F., Pareschi M.T. (2009). Spectral properties of volcanic materials from hyperspectral field and satellite data compared with LiDAR data at Mt. Etna. Int. J. Appl. Earth Obs. Geoinf..

